# Beyond the Hit: Muscle and Vascular Tissue Responses to Contact Exposure in Collision Sports—A Narrative Review

**DOI:** 10.1007/s40279-025-02296-1

**Published:** 2025-08-17

**Authors:** Craig Bolger, Jocelyn Mara, David B. Pyne, Andrew J. McKune

**Affiliations:** 1 University of Canberra Research Institute for Sport and Exercise, Kirinari Street, Bruce, Canberra, ACT 2617 Australia; 2 ACT Brumbies Rugby, Canberra, ACT Australia; 3 School of Health Sciences, Biokinetics, Exercise and Leisure Sciences, University of KwaZulu-Natal, Durban, KZN South Africa

## Abstract

Contact events in rugby codes such as tackling, running into contact, scrums, rucks, and contact with the playing surface may expose athletes to muscle damage known as impact-induced muscle damage. These repetitive impacts to muscle tissue have the potential to diminish muscle force production and delay recovery following contact-focused training and match-play. Repetitive exposure to contacts may also affect the surrounding vascular and neuronal tissues, an area that has received little attention in the collision sports. Depending on the severity and duration of tissue damage, repetitive contact exposure without sufficient recovery or noticeable adaptation may predispose collision sport athletes to impaired performance and long-term health complications. The aim of this narrative review is to provide a conceptual framework for understanding the physiological implications of contact exposure in collision sports. We examine the current understanding of impact-induced muscle damage (IIMD), how it differs from exercise-induced muscle damage (EIMD), and its relationship with contact exposure in collision sports. Drawing on both experimental animal models of contusion injury and limited human observational research, we explore the effects of repeated contact exposure on the microvasculature and its implications for both athletic performance and player welfare. To account for all tissues that may be affected by impacts, including muscle, nerve, vascular, connective tissue, skin, other organs and bones, we recommend impact-induced tissue damage (IITD) as the preferred descriptor rather than IIMD. Finally, we discuss the concept of contact adaptation and provide recommendations for future research on IITD in collision sports.

**Supplementary Information:**

The online version contains supplementary material available at 10.1007/s40279-025-02296-1.

## Key Points

**Table:** 

Despite similarities in the regeneration and remodelling process, IIMD may be more comparable to contusion injuries, characterised by the immediate onset of pain, a more severe inflammatory response, and a prolonged recovery period than typically observed in EIMD.
Evidence from experimental animal models of contusion injury and limited human data suggests that repeated contact exposure can compromise capillary networks and delay recovery. This may have implications for both acute performance and long-term player welfare outcomes.
The notion of ‘contact adaptation’ within collision sports remains largely unsubstantiated. There is currently limited empirical evidence to support this concept, particularly tissue-level adaptations.

## Introduction

### Background

In 2021, World Rugby surveyed nearly 600 participants across 18 elite men’s and women’s competitions to identify common contact training patterns in rugby union [1]. Based on their findings, best practice contact-load guidelines were developed alongside a review of recent injury data; however, the specific surveillance studies informing this review were not cited in the publicly available documentation. While these guidelines offer valuable recommendations for practitioners, limited consideration is given to the physiological responses to contact exposure. More recently, contact volume practices and perceptions in rugby league have been detailed [2]. Coaching staff were identified as the primary planners of contact volume, monitored through session duration. Perceptions of recovery time needed varied between players and staff, and even within staff groups, with coaches reporting shorter recovery periods between full-contact sessions compared to performance and medical staff [2]. These divergent perspectives among key stakeholders were further highlighted in another study [3]. Coaches prioritised tactical outcomes and supported higher contact volumes, whereas physiotherapists and strength and conditioning coaches emphasised structured progression, extended recovery, and advocated for periodised contact exposure. This inconsistency underscores the need for further objective analysis of the relationships between contact volume and recovery.

Contact events like tackling, carrying into contact, rucks, scrums, and mauls, coupled with secondary impacts with the ground and the subsequent impact of athletes falling onto the tackler or ball-carrier, may result in tissue damage at or adjacent to the site of impact [4]. Throughout a rugby union match, a team executes on average a total of 22 scrums, engages in 116 rucks, and performs 156 tackles [5]. These numbers vary substantially due to numerous contextual variables, such as rugby code, sex, positional role, tactical approach, opposition quality and level of competition [5–9]. Moreover, data collection method (GPS, instrumented mouthguard, video analysis), and the definitions and/or thresholds used to classify contact events introduce further variability [10–12].

Quantifying the contact demands in rugby codes remains challenging, and the forces that athletes are exposed to vary substantially. Initial investigations using Tekscan sensors embedded within shoulder pads showed that peak impact forces in male rugby union athletes ranged from ~ 470 to 2800 N, or 1.4 times (range 0.5–2.7) body mass (BM) during a front-on shoulder tackle [13]. However, given the stationary movement of the ball-carrier, these forces were likely underestimated. Recently, studies have enhanced the realism of rugby tackle simulations using a custom-built tackle simulator and a moving tackle bag with embedded force transducers [14]. Under these conditions, the tackler experienced forces nearly 3.5 times BM during dominant shoulder tackles. These forces were further pronounced when the simulated ball-carrier (tackle bag) is brought to the ground (4000–5000 N or 4–6 times BM) [15]. Repeated exposure to impact forces of this severity has the potential to cause damage to tissue, impairing muscle integrity, disrupting biochemical homeostasis, and triggering acute inflammatory responses which upregulates whole-body protein turnover [16–20]. Such homeostatic disruptions are likely energetically costly, contributing to increased post-exercise metabolism, further delaying the recovery time in collision sports compared to non-collision-based sports [21, 22]. Metabolomic analyses of elite rugby union players revealed a post-match shift toward gluconeogenesis persisting for 48 h [23]. This metabolic shift likely reflects increased energy demands during recovery, potentially exacerbated by insufficient carbohydrate intake. In this context, amino acid catabolism may be upregulated to support glucose production, further contributing to muscle tissue breakdown. Researchers now refer to this additional source of muscle damage as impact-induced muscle damage (IIMD) [4, 24]. For this review, the term ‘impacts’ will be used to encompass all physical impacts, while ‘contacts’ will specifically describe impact events in collision sports.

IIMD research in collision sports has mainly focused on relationships between contacts and subsequent neuromuscular, biochemical and/or perceptual responses [19, 25, 26]. Despite the interplay between muscle tissue and the microvasculature, there are limited data on vascular responses to impact exposure. Microvascular cells, second only to myofibres, provide a robust microvascular blood supply to support the metabolic demands of myofibres [27]. This dense interconnected network of skeletal muscle microvessels may be affected by compressive forces experienced during contact events. Given the limited evidence in collision sports, the vascular effects of contacts are primarily inferred from experimental animal contusion models and trauma research. Preliminary observations from rodent injury models indicate clear structural changes to the microvasculature following contusion injuries [28–32]. Traumatic brain injury (TBI) research also offers insights into secondary damaging mechanisms that could exacerbate the initial vascular damage caused by repetitive contact exposure [33–37].

The aim of this narrative review is to evaluate the current body of research investigating the physiological consequences of contact exposure in collision sports with a focus on rugby codes, specifically, rugby union. We address three key areas related to contact exposure: muscle response, vascular response, and tissue adaptation to repeated contact exposure. First, we provide an overview of IIMD, differentiating it from exercise-induced muscle damage (EIMD). Secondly, we review research exploring the relationships between contact exposure and acute physiological responses in collision sports. Thirdly, we examine the effects of repeated contact exposure on microvasculature, including potential relationships between vascular dysfunction and concussive and non-concussive impact exposure. Lastly, we analyse the concept of ‘contact adaptation’, and offer recommendations for future research in this area.

### Methodological Approach

A computerised literature search was conducted using PubMed from its earliest record through to June 2024, and again to May 2025. The search strategy combined rugby-specific terms (‘Rugby Union’ ‘Rugby League’ ‘Rugby Sevens’), with terminology related to contact events (‘tackle’ ‘collision’ ‘impact’), associated physiological responses (‘tissue damage’ ‘muscle damage’ ‘inflammation’ ‘biomarkers’) and measures of fatigue and recovery (‘subjective soreness’, ‘neuromuscular performance’). Additional searches explored vascular responses (‘vascular damage’ ‘microvascular’ ‘endothelial dysfunction’ ‘glycocalyx’ ‘cardiovascular’), as well as contusion injuries using controlled laboratory methods (‘drop-mass models’). Manual screening of reference lists was used to identify relevant papers covering EIMD, IIMD, concussion/TBI, contusion pathophysiology, glycocalyx and cardiovascular function. As this is a narrative review, we did not conduct a comprehensive systematic search with explicit inclusion/exclusion criteria, quality appraisal or bias analysis. The PubMed search strategy queries are provided in Online Supplemental Material (OSM) Resource 1.

## Pathophysiology of Muscle Damage in Collision Sports

### Exercise-Induced Muscle Damage

Exercise-induced muscle damage (EIMD), a typical response to unaccustomed and/or high-intensity exercise, is characterized by structural changes, including sarcomere, cytoskeletal and membrane damage [38]. Rapid or repeated muscle lengthening (eccentric) contractions, commonly encountered in activities like grappling, change of direction and high-speed running, can damage myofibrils, leading to EIMD [39]. Clinical signs and symptoms of myofibrillar damage include a temporary loss of muscle force and power, swelling (oedema), transient loss of range of motion, and systemic efflux of muscle-bound proteins, such as creatine kinase (CK) and myoglobin (Mb) into the systemic circulation, followed by the onset of delayed muscle soreness [16]. Mechanical strain experienced during eccentric muscle actions causes sarcomeres to overstretch beyond the point of myofilament overlap, leading to ‘popped sarcomeres’ [40]. This theory stems from early observations showing Z-disk disorganisation, widening of perimysial spaces between fascicles, and separation of myofibres following maximal eccentric contractions [41–43]. Other well-supported theories attribute the acute loss in strength (1–3 days) following eccentric contractions to excitation–contraction (E–C) uncoupling [44–46], while strength loss persisting beyond 3 days is thought to result primarily from the damaged contractile proteins [47].

Following acute trauma to muscle tissue, often referred to as primary damage, a secondary enzymatic response is thought to induce further damage to otherwise uninjured muscle tissue [17, 48, 49]. Initial myofibrillar disruption causes an influx of calcium (Ca^2^⁺) that enters the cytosol through stretch-activated channels and/or permeable sections of the damaged sarcolemma [16]. Elevated intracellular Ca^2^⁺ activates calpain, a protease that degrades structural proteins critical for contraction and myofibrillar integrity, contributing to EIMD [16].

Activated immune and inflammatory cells, such as neutrophils and macrophages, migrate to sites of tissue damage to support repair and remodelling processes. Neutrophils are among the first responders, clearing cellular debris and promoting a pro-inflammatory response through the secretion of cytokines like tumour necrosis factor-alpha (TNF-α) and interleukin-1 (IL-1) [50]. These cytokines are essential in mediating the inflammatory response, facilitating cellular communication, and amplifying the pro-inflammatory cascade. This, in turn, promotes the migration of additional pro-inflammatory cells to the site of injury, aiding in the phagocytosis of damaged tissue. Within 4–24 h, pro-inflammatory M1 macrophages are attracted to the damaged site, where they further contribute to the secretion of pro-inflammatory cytokines and engage in phagocytosis of damaged muscle tissue [51, 52]. Neutrophils and M1 macrophages interact to regulate this early pro-inflammatory phase, which typically lasts for 24–48 h, depending on the severity of the EIMD [51].

Subsequently, anti-inflammatory cells including M2 macrophages and T-regulatory lymphocytes, migrate to the damaged site and dampen the pro-inflammatory cellular response. Phagocytosis of cellular debris drives this phenotypic shift from pro- to anti-inflammatory macrophages, facilitating the resolution of tissue inflammation following exercise. M2 macrophages secrete cytokines like IL-10, which promote myoblast proliferation, differentiation, and expansion of the satellite cell pool [51]. This sequence of both pro- and anti-inflammatory events is crucial for the removal, regeneration, and remodelling of damaged tissue, and plays a vital role in the adaptive tissue process to reduce or mitigate potential muscle damage from future exposure to similar exercise bouts [16]. This concept of secondary damage/strength loss has been challenged: a meta-analysis of 223 human and animal studies found no secondary reduction in strength in the 1–3 days after acute muscle injury, instead showing steady recovery [53]. However, this does not preclude secondary cellular damage. Approximately 50–75% of the initial post-eccentric strength deficit is due to E–C uncoupling, which resolves rapidly (1–3 days), whereas the remainder, reflecting structural disruption, recovers more slowly. As fibres regain force via E–C repair, inflammation-mediated damage in adjacent tissue may continue, yet remain undetected in whole-muscle strength assessments [53]. For a more comprehensive exploration of EIMD, readers are directed to several comprehensive reviews [54–58].

### Impact-Induced Muscle Damage

**Fig. 1:**
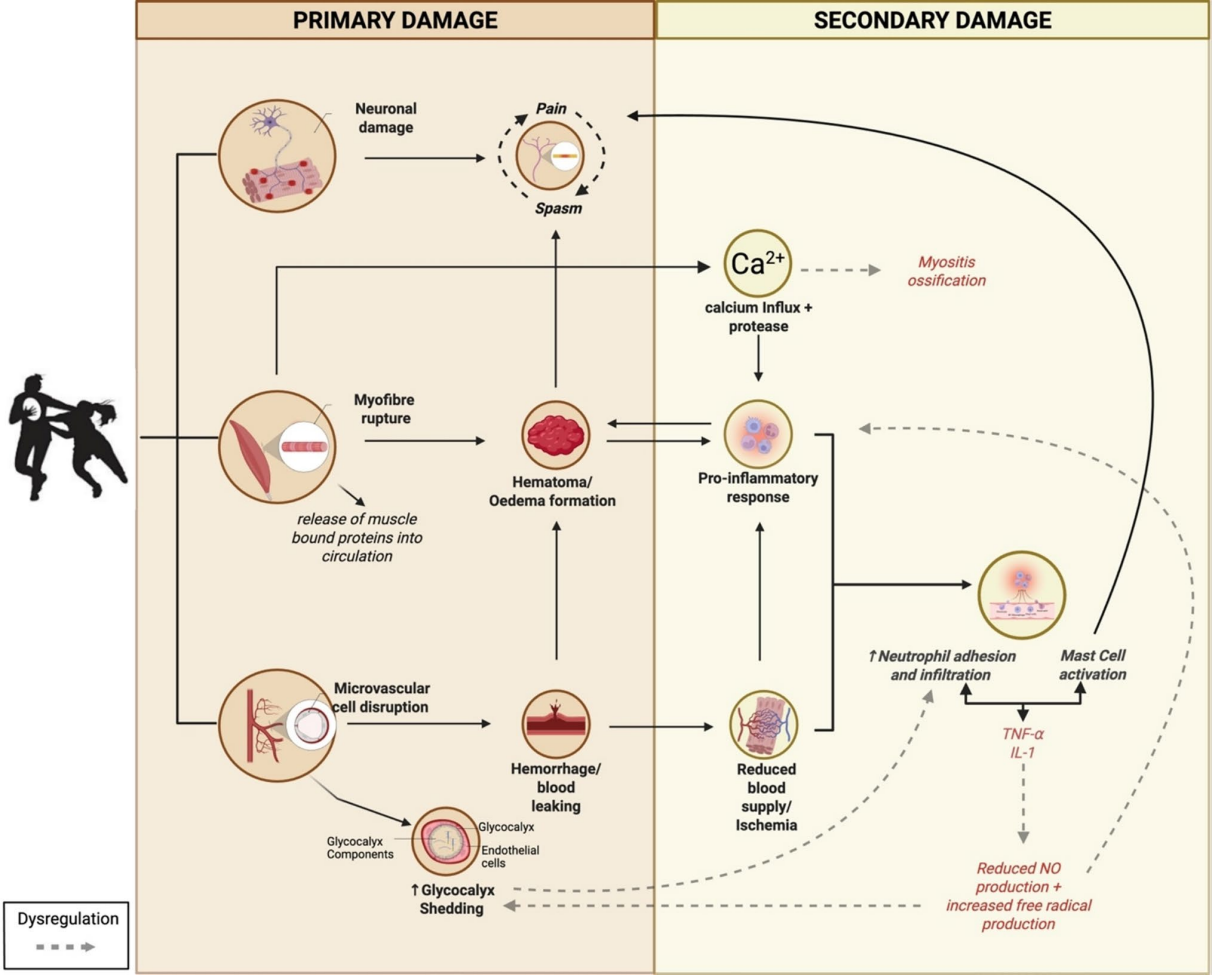
Illustration of the flow of primary and secondary tissue damage following impact-induced tissue damage (IITD). Grey dashed lines represent pathways that may contribute to excessive tissue damage under conditions of high levels of IITD without adequate recovery. *TNF-α* tumour necrosis factor-alpha, *IL-1* interleukin-1, *NO* nitric oxide, *Ca*^*2*^*⁺* calcium. Created in BioRender. Bolger, C. (2025) https://BioRender.com/i99q395

One of the defining aspects of collision sports such as rugby union is the frequency and magnitude of the contacts that athletes are exposed to during match-play [25, 59]. Like EIMD, repeated exposure to contacts throughout match-play and training can cause damage at or adjacent to the site of impact eliciting IIMD [4]. Direct damage to muscle tissue can reduce muscle force production and impair muscle integrity [60]. The regenerative and remodelling process of muscle tissue that follows is likely energetically demanding, potentially raising post-exercise metabolism and resting metabolic rate, caused by increased whole-body protein turnover in response to muscle damage [21]. Similar to the recovery dynamics observed after EIMD, the temporal disruption of muscle force-generating capacity after IIMD is intricately linked to the frequency and intensity of contact exposures [25, 61]. Despite the commonalities in regeneration and remodelling processes, IIMD likely differs with an augmented inflammatory infiltration and subsequent secondary damage response (Fig. 1). Research on contusion injuries reveals a more pronounced and prolonged activation of muscle protein degradation pathways compared to the catabolic responses typically observed after exhaustive exercise, suggesting a more severe and sustained muscle breakdown process [62].

While the inflammatory processes following EIMD and tissue strains share common mechanisms, such as leukocyte infiltration and cytokine production [63], the inflammatory process in EIMD is generally well regulated and balanced, facilitating effective muscle repair and adaptation [51]. In contrast, muscle strains result in a secondary pro-inflammatory response that is more severe and less controlled than that observed following EIMD [64]. Muscle strains involve sudden fibre tearing from a tensile load, causing immediate symptoms and slow, often incomplete recovery with a high risk of recurrence. In contrast, EIMD occurs gradually during eccentric exercise, with delayed symptoms and a repair process that strengthens the muscle against future damage [64]. It is unclear if IIMD is to contusion injuries what EIMD is to strain injuries, where EIMD and strain injuries are distinctly different entities. Clinical outcomes such as the immediate onset of symptoms following impact suggest that IIMD may be more akin to contusions. If true, the pro-inflammatory response could be more severe and uncontrolled, potentially leading to a longer recovery in IIMD than EIMD [17, 29, 63].

Following a muscle contusion injury, the myofiber membrane is compromised, resulting in an influx of extracellular Ca^2^⁺. This Ca^2^⁺ overload initiates muscle protein degradation, and in extreme cases myositis ossification [65]. Resident mast cells are rapidly activated and degranulate, releasing pro-inflammatory cytokines such as TNF-α, IL-1 and histamine, which recruit additional mast cells, neutrophils, and other immune cells to the injury site [66]. Simultaneously, resident neutrophils are activated and release pro-inflammatory cytokines, including TNF-α, IL-1β and interferon-γ. These cytokines facilitate the recruitment and infiltration of peripheral neutrophils into the extracellular space surrounding damaged muscle fibres within 1–4 h of the initial insult [51, 66].

Neutrophil infiltration may be prolonged following IIMD, contributing to secondary damage [58]. Neutrophils contribute to post-injury events in two ways: by phagocytosing damaged tissue and by amplifying the inflammatory process through the release of pro-inflammatory cytokines and free radicals [67, 68]. Although essential for early repair, phagocytosis and the neutrophil respiratory burst can exacerbate muscle damage and potentially harm surrounding healthy tissue if the response is prolonged [68]. Experimental depletion of neutrophils prior to ischemia–reperfusion decreased muscle damage by nearly 40% in mice, highlighting the role of neutrophils in secondary injury [69]. Additionally, IIMD may delay the M1-to-M2 macrophage transition due to more extensive trauma and vascular disruption compared to EIMD. Delays in the M1–M2 transition in contusions are associated with persistent M1 pro-inflammatory activity and impaired muscle regeneration [31, 70].

Tissue recovery may be further delayed following IIMD by tissue ischemia, where the blood supply to the damaged tissue is restricted. In contusion injuries, the primary damage involves muscle tissue damage and vascular injury, often leading to increased oedema and haematoma formation [18, 29]. Initial damage to blood vessels, blood clotting, increased blood viscosity, elevated extravascular pressure caused by bruising and muscle spasms, and swelling of cells may collectively contribute to tissue ischemia [18, 71, 72]. As a result, oxygen and nutrients are delayed in reaching the affected muscle tissue, which is thought to result in slower recovery [17, 31, 58].

The unique combination of contacts and high-intensity eccentric movements may exert a more pronounced effect on muscle damage and soreness than each factor in isolation [4]. This interplay between IIMD and EIMD, along with other psycho-physiological factors of fatigue, can potentially extend the overall recovery period following rugby codes and other collision sports [60, 73]. A more detailed understanding of the distinct contribution of each and how they interact is crucial for guiding the prescription of training.

### Contact Exposure and Tissue Damage

#### Match-Play, Contact Exposure and Muscle Tissue Damage

Eccentric muscle contractions are common in rugby codes, with forwards in rugby union frequently experiencing supramaximal eccentric/yielding isometric contractions while being driven backward during scrums [74–76]. These actions impose substantial stress on muscle tissue, with post-training or post-competition muscle damage to be expected [77]. Following rugby union match-play, high levels of muscle damage can reduce neuromuscular performance and increase perceptual fatigue and soreness, returning to baseline within 48–72 h [77]. Biochemical responses typically peak (120–450% of baseline values) between 12 and 24 h [60], but may persist beyond 72 h depending on various factors [60]. For example, elevations of indirect markers of muscle damage (CK) can stay elevated above baseline for up to 120 h [61].

Contrary to the well-understood running demands and subsequent muscle damage recovery timelines, the physiological repercussions of contacts in rugby codes have not been well characterized [4]. Contact events make up a very important part of the game, with the tackle, ruck and scrum forming the most common physical interactions in rugby union. This is important for forwards, who are typically heavier, engage in more physical contacts, and endure prolonged periods of static exertions compared to backs [5]. As a result, a team’s performance often hinges on their collective ability to endure these events to secure victory [78–80].

Early investigations shed light on the relationship between the frequency of contacts encountered by male rugby union players and biomarkers of muscle damage [25]. Large correlations were evident between the number of contacts, peak Mb (*r* = 0.85) and peak CK (*r* = 0.92), with peak levels observed 45 min (Mb = 980 μg/l) and 24 h (CK = 1081 U/l) post-match, respectively. Similar interactions between number of contacts and markers of acute muscle damage have been observed in subsequent studies [19, 24, 59]. For example, total contacts correlated moderately (*r* = 0.44–0.64) with CK concentrations [81]. These associations extend beyond biochemical markers of muscle damage. Studies show similar associations between the number of contacts and subsequent reductions in lower limb peak power and rate of force development, assessed using a counter movement jump (CMJ) [82], and subjective ratings of muscle soreness in the 24–72 h after match-play compared with baseline measures [83, 84]. Furthermore, total contact count was linked to decrements in upper-body neuromuscular performance. Conversely, despite pronounced declines in CMJ performance, some studies report no correlation between contact and CMJ performance [84]. Likewise, post-match testing (≤ 2 h) in rugby league players showed no link between contact exposure and isokinetic measures of maximal voluntary contraction [85].

Given the nature of contact exposure, upper-body neuromuscular function may be more sensitive to performance changes than that of the lower body [85]. Variations in outcome metrics, where peak power and rate of force development appear more susceptible to attenuation in performance than peak force, jump height or flight time, might explain differences [60]. Differences in measurement devices (e.g., OptoJump vs. force plates), and timing (2 h vs. 24–60 h post-match) may contribute to these inconsistencies [82, 84, 85]. Furthermore, univariate analyses may be limited by multicollinearity, reducing inference validity [86]. To address this, a recent study used multivariate analysis to model how a latent external load construct, combining contact and running metrics, predicted post-match CMJ fatigue [87]. Tackles, mauls and high-speed running were primary contributors to the forwards’ load construct but explained only 4% of their pre- to post-match CMJ variation. In contrast, backs showed a stronger relationship (32%), driven by high-speed running and accelerations, with attacking-ruck counts contributing marginally. Practitioners should consider monitoring upper-body neuromuscular fatigue in forwards, as it may be more sensitive to performance changes. Overall, contact exposure shows moderate to strong associations with muscle damage and perceived soreness or wellbeing, though links to neuromuscular performance are less consistent.

Three studies have examined the effects of contact exposure on neuromuscular fatigue, muscle damage, or perceived exertion in female rugby players, specifically two in rugby sevens and one in rugby union [26, 88, 89]. Following a 2-day Women’s Rugby Sevens tournament, moderate changes in muscle damage markers were observed, primarily attributed to high-speed running and impacts > 10 g, assessed via GPS-embedded accelerometers [26]. National players exhibited higher on-field activity in terms of total time, distance, high-speed running (> 5 m∙s^−1^) and impacts > 10 g, alongside smaller declines in performance compared to state players. Additionally, state players showed a greater CK response, with a fourfold increase (ΔCK = 737 U/L) compared to a twofold rise in national players (ΔCK = 502 U/L). It appears that athletes could become more accustomed to both EIMD and IIMD with training age and training exposure.

In another study, female sevens players reported impairments in wellbeing, perceived recovery, and muscle soreness across a 2-day tournament [89]. Contact exposure was moderately associated with muscle soreness (*r* = − 0.69), while high-intensity running correlated with elevated fatigue (*r* = − 0.60). Despite these disturbances in perceptual fatigue, no decrements in neuromuscular performance were detected. Finally, observations from 74 international sevens matches noted that both contact frequency and playing time predicted ~ 30% of post-match RPE variation (*R*^2^_adj = 0.31) [88]. Notably, the influence of playing time on RPE diminished as contact frequency increased, suggesting that physical contacts may be a primary driver of perceived player load. Collectively, these findings highlight contact as a key contributor to perceptual and biochemical fatigue, even in the absence of measurable performance impairments. Monitoring contact exposure may help inform recovery strategies following match-play.

#### Isolated Impact Exposures and Muscle Tissue Damage

There are a limited number of studies that have investigated the functional implications of isolated contact exposure and muscle tissue damage. These studies have typically investigated this question under controlled conditions that try to mimic the contact demands of rugby union or rugby league match-play. Investigators have examined the effect of contacts on muscle damage through small-sided games, intermittent sprint protocols, and team-based conditioning circuits [90–92]. Substantial reductions in neuromuscular performance, increased concentration of biomarkers of muscle damage, and increased perception of effort and muscle soreness, have been reported following contact versus non-contact activities. Other approaches have examined the psychophysiological and neuromuscular response following training involving contact and compared them to non-contact sessions where drills consisted of bodies in front of the ball-carrier [93–95]. Contact-focused sessions elicited greater upper-body neuromuscular and perceptual fatigue, and elevated CK, whereas non-contact sessions elicited higher running intensities and distance covered, manifesting as greater impairments in post-session CMJ performance [93]. Based on these observations, it appears that contact exposure leads to heightened deterioration in muscle function and, consequently, performance [90–92]. It is worth noting that the methodological approaches used in these studies typically involved high-intensity running and/or wrestling, which likely result in more work being performed [55, 96]. These methodological constraints have the potential to induce EIMD, making it difficult to isolate and determine the extent of damage arising from IIMD.

Tackling is a physically demanding activity involving multiple muscle groups, including the upper body musculature used for grasping, wrestling or restraining the ball-carrier to bring them to the ground and disrupt the passage of play. These movements can often lead to the development of EIMD and increased energy expenditure [96]. A recent systematic review reported higher total energy expenditure in contacts versus non-contact-based activities [21]. The increased energetic cost of contacts may stem from increased whole-body protein turnover due to IIMD, which demands more energy to return to baseline measures [97]. However, the specific causal mechanisms behind the observed increase in total energy expenditure remain unclear and could relate to genuine IIMD resulting from compressed muscle tissue and subsequent tissue regenerative processes, and/or extra work performed during muscular contractions.

#### Drop-Mass Models in Humans

Only two studies have sought to differentiate IIMD from EIMD in humans by employing a laboratory-based drop-mass model to induce muscle damage [18, 98]. In one study, a flat metallic and rubber-padded plate was placed on a Smith machine and repeatedly dropped on the participants’ anterior, lateral and posterior thigh regions [98]. Participants experienced reductions in jump height and sprint performance for up to 48 h post-impact and increased perception of muscle soreness up to 72 h. However, there were no noticeable changes in Mb, suggesting that IIMD might not have been evoked and changes in performance and perceived muscle soreness could relate to another form of fatigue [98]. Two alternative explanations for the absence of changes in Mb include the characteristics of the impacting object and/or the lower intensity of impacts used. Flat-surfaced weights distribute force uniformly, while a hemispherical impactor, more akin to a shoulder, concentrates force centrally, increasing injury severity [58, 99]. Secondly, experimentally evoking sufficient muscle damage to measure biomarkers without causing undue pain, discomfort and short-term decrements in performance is challenging. The lower impact forces used in this study compared to forces experienced during an active shoulder tackle (~ 900–1100 N vs. 4000–5000 N) may not have exceeded the tissue’s tolerance [15]. The padding between participants’ legs and the absence of ground impact post-contact may have further reduced total muscle damage. These methodological limitations restrict the conclusions that can be drawn about IIMD from this model.

A second drop-mass model used a weighted object with a spherical, hard resin head eliciting varying degrees of oedema, muscle soreness, and decrements in muscle performance [18]. Unsurprisingly, the heaviest object (7.2 kg) induced greater oedema, voluntary force loss, pain and elevated serum CK compared to lighter loads, with CK levels remaining fivefold above baseline at 72 h post-impact. The delayed CK response, which was undetectable at 30 min, was attributed to secondary rather than primary muscle damage. However, concluding that no primary damage occurred based on this observation seems questionable. It is well documented that CK has a delayed kinetic response, peaking 24–48 h after tissue damage. In severe cases, like crush injuries, serum CK may rise as early as 2–12 h post-trauma [100]. Yet, even in these extreme cases, immediate elevations in CK are not observed. This delay is partly due to the large molecular size of CK (CK = 86 kDa vs. Mb = 17 kDa) [101]. As a result, CK is transported by the lymphatic system, delaying release from the damaged muscle tissue into the systemic circulation [102, 103]. Another limitation was the use of a single impact, which does not replicate the repetitive contacts typical in collision sports. Muscle damage can result from excessive peak force, cumulative loading, or rapid tissue deformation, all important considerations when designing experimental IIMD protocols.

Future studies should employ either statistical-shape modelling to develop an object of similar shape to a shoulder, or a hemispherical impactor, with compliant outer layers, to better replicate the loading mechanics and tissue deformation patterns typical of shoulder tackles in rugby. Impacts should reflect rugby-specific forces (1.5–4.5 kN) and include repeated strikes to mimic gameplay. Combining early (1–6 h) and delayed (24–72 h) biomarker sampling with imaging will improve characterisation of damage kinetics.

While isolated muscle contusion models are a valid and necessary means of exploring IIMD, their findings may not fully translate to real-life contact scenarios. Therefore, a combination of contusion models and tackle-based investigation studies is needed to address this complex issue. A well-designed study that controls for non-contact-based efforts and compares the ball-carrier (involving limited grasping/muscle contractions, but potentially greater tissue compression) with the tackler (involving greater physical exertion, but potentially less tissue compression) offers a more ecologically valid approach for investigating IIMD. Such research could provide practitioners with greater insight into which contact event, i.e., tackling versus ball carries into contact, has a larger effect in the context of IIMD.

## Vascular Damage Following Contact Exposure

The vascular system is crucial for both long-term player welfare and athletic performance, with the endothelium, particularly the glycocalyx layer, playing a key role in maintaining vascular health. To our knowledge, no research has investigated the effects of contact exposure in collision sports on vascular structure or function. The following presented insights are based on experimental animal contusion models and trauma studies. We also present contact exposure in the context of concussive and non-concussive head trauma, exploring potential mechanisms that may contribute to systemic vascular dysfunction in collision sport athletes following head trauma.

### More Than Muscle Damage: A New All-Encompassing Term

The vascular system plays a critical role in muscle function, recovery and adaptation, contributing to enhanced physical performance [104]. The vascular system serves two primary functions: (i) distributing blood throughout the body, and (ii) facilitating the exchange of materials between the vascular space and tissues, to meet the metabolic demands of a given activity. Improved vascular function can enhance exercise performance by increasing delivery of oxygen and nutrients to muscles, improving the exchange of materials between the vascular space and tissues, enhancing endurance, reducing fatigue, and promoting faster recovery and training adaptations [104]. Moreover, vascular function is closely linked to long-term cardiovascular health. A healthy vascular system is essential for maintaining normal blood pressure, preventing development of cardiovascular diseases such as hypertension, atherosclerosis and heart failure [105]. Impaired vascular function can reduce exercise tolerance, increase the risk of cardiovascular events, and compromise overall health, underscoring its importance for medical and performance professionals working with athletes [106].

Tissue damage from direct compression can evoke immediate ultrastructural changes or secondary enzymatic damage to muscle, connective tissue, nerves and blood vessels [17]. Vascular trauma following impact-induced tissue damage (IITD) during exercise and its subsequent recovery timeline has received limited attention within the sport and exercise science research community. The term IITD encompasses all tissues that may be affected by impacts, including muscle, nerve, vascular, connective tissue, skin and other organs, and may therefore be a more preferred descriptor than IIMD (Fig. 1). Understanding the potential IITD and the recovery dynamics of the vascular, specifically microvascular, network is crucial for performance staff, whose responsibility is to enhance performance while minimising excessive training loads.

### Overview of Endothelial Function, Exercise Performance and Player Welfare

#### The Endothelium

It is well established that regular exercise improves vascular function, enhances cardiovascular performance, and reduces cardiovascular risk in both healthy and clinical populations. Endothelial cells lining the luminal surface of blood vessels play a pivotal role in vascular function and overall cardiovascular health [107]. The endothelium acts as a barrier between the blood and underlying tissues, modulating blood flow, and producing vasoactive hormones, like nitric oxide (NO), which promote vasodilation via smooth muscle relaxation [108, 109]. Preserving the functional integrity of the endothelium is crucial for maintaining adequate blood flow to muscle tissue, upholding cardiovascular health, improved recovery from training, and enhanced performance. Pathological changes in the structure and function of the endothelium, whether due to aging or excessive exercise, can impair vascular function [110, 111]. A dysfunctional endothelium, characterised by increased vasoconstriction, vessel stiffness and vascular permeability, serves as an early sign of atherosclerosis and a predictor of cardiac risk [110]. These relationships underscore the importance of vascular health in physical performance and player welfare.

#### The Endothelial Glycocalyx

A key regulator of endothelial function is the endothelial glycocalyx (eGC). The eGC is a micro-thin protective gel lining on the interior surface of blood vessels which helps maintain endothelial function, arterial elasticity and healthy blood pressure [112, 113]. Many researchers now regard the eGC as one of the first lines of defence against vascular diseases, and damage imposed to the eGC is considered an early marker of endothelial dysfunction [112, 114]. Factors such as sepsis, trauma, ischaemia/reperfusion, and prolonged hyperglycaemia contribute to eGC shedding, detectable via blood biomarkers. Persistent eGC damage is linked to endothelial dysfunction, increased vascular permeability, and impaired oxygen and nutrient delivery [113, 115, 116].

High-intensity exercise can increase the shedding of eGC components such as syndecan-1, heparan sulfate and hyaluronan into the systemic circulation [117]. For example, changes in eGC shedding after 4 × 4 min of running and cycling were observed, with increases approximating 7.8% in syndecan-1 levels, 21% in heparan sulfate, and 44% in hyaluronan [117]. The extent of this eGC shedding post-exercise is influenced by factors including exercise intensity, duration and training age [114, 117]. Some evidence suggests exercise can have a protective effect on eGC shedding. Moderate-intensity endurance training can improve eGC layer integrity, lowering levels of circulating eGC damage markers and enhancing antioxidant defence [118]. Increased antioxidative capacity, a typical training adaptation, may help mitigate exercise-induced vascular damage, and promote long-term cardiovascular health [119].

#### Endothelial Function and Excessive Exercise

Training adaptations may be dose-dependent, with some research indicating that specific modes of exercise and/or excessive training change arterial stiffness and impair vascular function [120, 121]. One study in female track athletes reported negative changes in vascular function following pre-season training [111]. Athletes presented with reduced flow-mediated dilation, a non-invasive measure of vascular function, and elevated serum concentrations of hyaluronan and syndecan-1. These changes were accompanied by lower serum testosterone and free testosterone, elevated cortisol concentrations, a twofold increase in CK (indicating greater skeletal muscle stress), and reduced levels of alpha-1-acid glycoprotein, a proposed marker of non-functional over-reaching [122]. While these impairments may be temporary, their recovery timelines remain unclear.

Variable outcomes relating to the effects of exercise on biomarkers of vessel damage are likely attributed to differences in total training volume and intensity. Training programs performed by athletes at the domestic and international level differ markedly from experimental protocols, with professional track athletes completing up to 9-times more training compared to high-intensity experimental studies [111]. Similarly, a typical training week for domestic rugby union can amount to 8–12 h of combined contact training, resistance exercise, conditioning, sprint and team training sessions, almost three to six times the training hours compared to experimental studies [123]. The current training load placed on elite rugby union athletes, along with the variability of exercise modalities they are exposed to, could elicit similar cardiovascular outcomes in the female track athletes. With appropriate load-monitoring strategies and planned reductions in training volume, it may be possible to mitigate adverse vascular adaptations [124]. Further research is warranted to understand the rate at which these markers return to baseline following acute exercise, enabling performance staff to make informed, evidence-based decisions that protect vascular integrity.

### Vascular Response to Impact-Induced Tissue Damage (IITD)

#### Primary Damage

IITD may be present in the peripheral microvasculature following contact exposure. Evidence from a drop-mass model inducing muscle contusion in the hind limb of mice revealed disrupted myofibers and non-flowing capillaries within the affected tissue [28]. This is consistent with other experimental models of myofiber and microvascular injury, which employed either myotoxins or punch biopsies to induce tissue damage [125, 126]. Intramuscular injection of the myotoxin BaCl₂ resulted in capillary fragmentation and myofiber degeneration in mice, yielding a disorganised and sub-optimal microvascular regrowth over 5–10 days and full recovery only after 21 days [125]. This delayed recovery of damaged myofibers and microvasculature negatively influences the efficiency of the signalling mechanisms that regulate oxygen supply between the muscle tissue and its capillary network [127]. Although these experimental injuries are more severe and focal than repeated contact exposure during match-play, they illustrate how IIMD could disrupt and prolong microvasculature recovery.

Disrupted capillary networks can negatively affect muscle oxygenation by increasing diffusion distances and compromising tissue oxygen delivery [128]. It is conceivable that mechanical forces experienced during contacts could disrupt structural components of the blood vessels such as the eGC, with the degree of degradation highly correlated with the severity of IITD [113]. Evidence from a drop-mass model on rats showed that severe blunt trauma impairs distal microvascular regulation and oxygenation, likely a result of endothelial injury [72]. In another study, a severe crush injury resulted in substantial vascular damage, increased production of inflammatory cytokines, and heightened inflammation and fibrosis [29]. In contrast, a mild contusion preserved vascular integrity in the skeletal muscle of the mouse hind leg. However, this study was limited to a single impact event; thus, repeated mild contusions/impacts could lead to progressively greater vascular damage.

Caution is warranted when interpreting findings from rodent models. Differences in injury volume, impact force, the use of involuntary muscle force assessments in anaesthetised rodents, methods of inducing tissue damage, and inherent physiological and genetic differences between humans and rodents, can limit the translation of findings [129].

#### Secondary Damage

Secondary vascular damage is likely mediated by the interaction between neutrophils and the eGC. While neutrophils play an important role in the inflammatory response, tissue injury may arise when their activity becomes excessive or dysregulated [50, 130, 131]. Damage to the microvasculature, as well as larger macro-vessels such as conduit arteries, can result from such secondary inflammatory processes. While the acute inflammatory response following IITD is essential for clearing damaged cells and initiating tissue repair [33], continued training without sufficient recovery may excessively activate the innate immune system, compounding tissue damage and impairing subsequent repair and remodelling [17, 132].

**Fig. 2:**
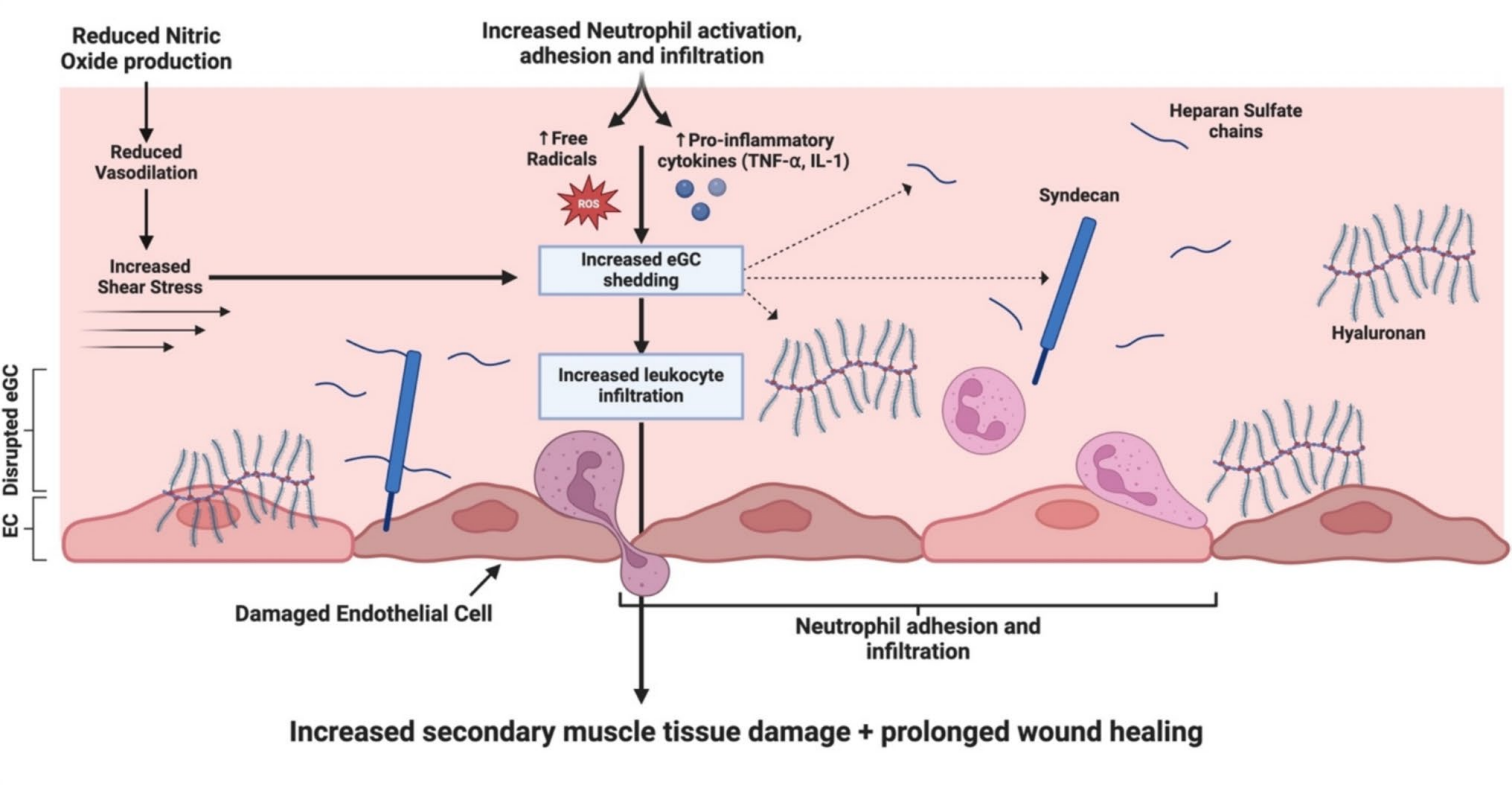
Proposed mechanism for endothelial glycocalyx (eGC) degradation following excessive impact-induced tissue damage (IITD): The structural integrity of the eGC relies on a balance between enzymatic degradation and de novo biosynthesis of its components, as well as the adsorption of circulating molecules from the blood. Several enzymes responsible for eGC degradation are activated by pro-inflammatory cytokines, such as tumour necrosis factor-alpha (TNF-α) and interleukin-1 (IL-1), along with reactive oxygen species (ROS), which promote the damage and shedding of key glycocalyx components. This process leads to the release of components like heparan sulfate, hyaluronan, and syndecan into the circulation. As the eGC degrades, its structural integrity diminishes, leading to a thinner layer that increases leukocyte adhesion to the endothelial surface. This promotes excessive neutrophil infiltration into surrounding tissues, further exacerbating injury in previously unaffected areas. Conditions characterized by excessive loss of the eGC may also exhibit reduced endothelial nitric oxide (NO) production, which enhances neutrophil adhesion and perpetuates vascular dysfunction and inflammation. Created in BioRender. Bolger, C. (2025) https://BioRender.com/y99y354

Pro-inflammatory mechanisms can further degrade the eGC via the activation of enzymes such as metalloproteinases, heparanase and hyaluronidase [113]. These enzymes are activated by free radicals and pro-inflammatory cytokines such as TNF-α and IL-1β, both of which are released by neutrophils during the initial inflammatory response. As the eGC deteriorates, its structural integrity weakens, increasing leukocyte adhesion to the endothelium and promoting excessive neutrophil infiltration into surrounding tissue, thereby exacerbating injury to previously unaffected areas [131, 133] (Fig. 2).

The eGC influences neutrophil adhesion via regulation of endothelial-derived NO production [131], a process medicated by shear stress-induced mechanotransduction [134]. NO suppresses the expression of adhesion molecules which facilitate leukocyte binding to the vascular endothelium [131]. Physiological states marked by excessive loss of eGC may impair NO production, increasing neutrophil adhesion and infiltration [135]. In rat models, systemic administration of l-arginine, a substrate for NO synthase, reduces neutrophil-endothelial adhesion and tissue damage when given prior to reperfusion of an occluded limb, highlighting the protective role of NO in preserving vascular integrity during the initial inflammatory response [136]. Several direct and indirect methods have been used to assess eGC integrity and degradation. Indirect approaches include measuring eCG biomarkers such as syndecan-1, hyaluronan and heparan sulfate. However, the non-specificity of these biomarkers in quantifying damage and potential interference from other cell surfaces is a limitation [137]. Direct methods like intravital and electron microscopy provide high-resolution imaging but are mainly limited to animal or ex vivo studies [112, 138]. A promising compromise for human research is sidestream dark-field imaging, a non-invasive technique that estimates eGC thickness via the perfused boundary region of sublingual microvessels [139]. Combining biomarker analysis with sublingual imaging could offer a practical and informative approach to monitor eGC damage.

In collision sports, athletes are exposed to multiple contacts across a training week, often with no more than 2 days between sessions. Such a short recovery time might be insufficient for the microvasculature to return to normal, leading to a chronically disorganised and disrupted capillary network. Consequently, the eGC may not have sufficient time to recover, resulting in excessive activation and shedding of structural proteins [111, 113]. If eGC integrity is compromised by repeated bouts of contact-exposure, investigators could explore interventions aimed at preventing endothelial dysfunction. One promising avenue may involve administering or supplementing with structural components of the eGC to mitigate damage and preserve vascular health [140, 141].

### Traumatic Brain Injury and Systematic Vascular Dysfunction

In collision sports, the risk of structural and functional alterations to the brain, both acute and cumulative, warrants consideration within the broader framework of IITD. Even in the absence of clinical symptoms, athletes may experience subtle but meaningful changes to the brain following repeated non-concussive head acceleration events (HAEs) [142–146]. In rugby union, most contact events do not involve direct head contact between the tackler and ball-carrier [147]; however, indirect HAEs are common and can exceed biomechanical thresholds previously associated with clinically diagnosed concussion [148].

HAEs cause the brain to shift within the skull, leading to transient compression against the cerebrospinal fluid and, at higher magnitudes, brief contact with the inner surface of the cranium. Rapid rotational forces induce shear stress across brain tissue, contributing to structural deformation and potential cellular damage [149]. Over time, cumulative exposure to HAEs may elevate the risk of future concussion and neurodegenerative disease [150–152]. Lifetime HAE exposure in American Football players has outperformed years of play and reported concussion history in predicting chronic traumatic encephalopathy pathology and neuropsychological impairment [145, 153].

Head trauma, which may be considered a severe form of IITD, occurs when an impact with another person or object generates biomechanical forces that are transmitted to the brain, resulting in transient neurological impairment [154]. Head trauma can range from non-concussive events to TBI, with TBI classified into mild, moderate or severe categories based on clinical criteria, where mild TBI is synonymous with concussion [154–156]. A non-concussive event occurs when a direct blow to the head or an indirect HAE does not produce noticeable clinical symptoms [142, 157]. In contrast, TBI typically leads to neurological dysfunction, with symptoms that vary depending on the severity of the TBI [156]. Head trauma, regardless of severity, can lead to increased levels of circulating free radicals and reduce vasodilatory metabolite production such as NO, resulting in systemic vascular dysfunction [33, 34]. In a mouse model, TBI resulted in microvasculature endothelial dysfunction which persisted for at least 24 h post injury [34]. Furthermore, TBI induced long-lasting endothelial dysfunction in the aortic arteries of mice [36]. Finally, formerly concussed retired rugby players showed reduced systemic NO bioactivity, though vascular function was not assessed [158]. These adverse systematic vascular consequences may not be limited solely to TBI [35, 37, 116].

**Fig. 3:**
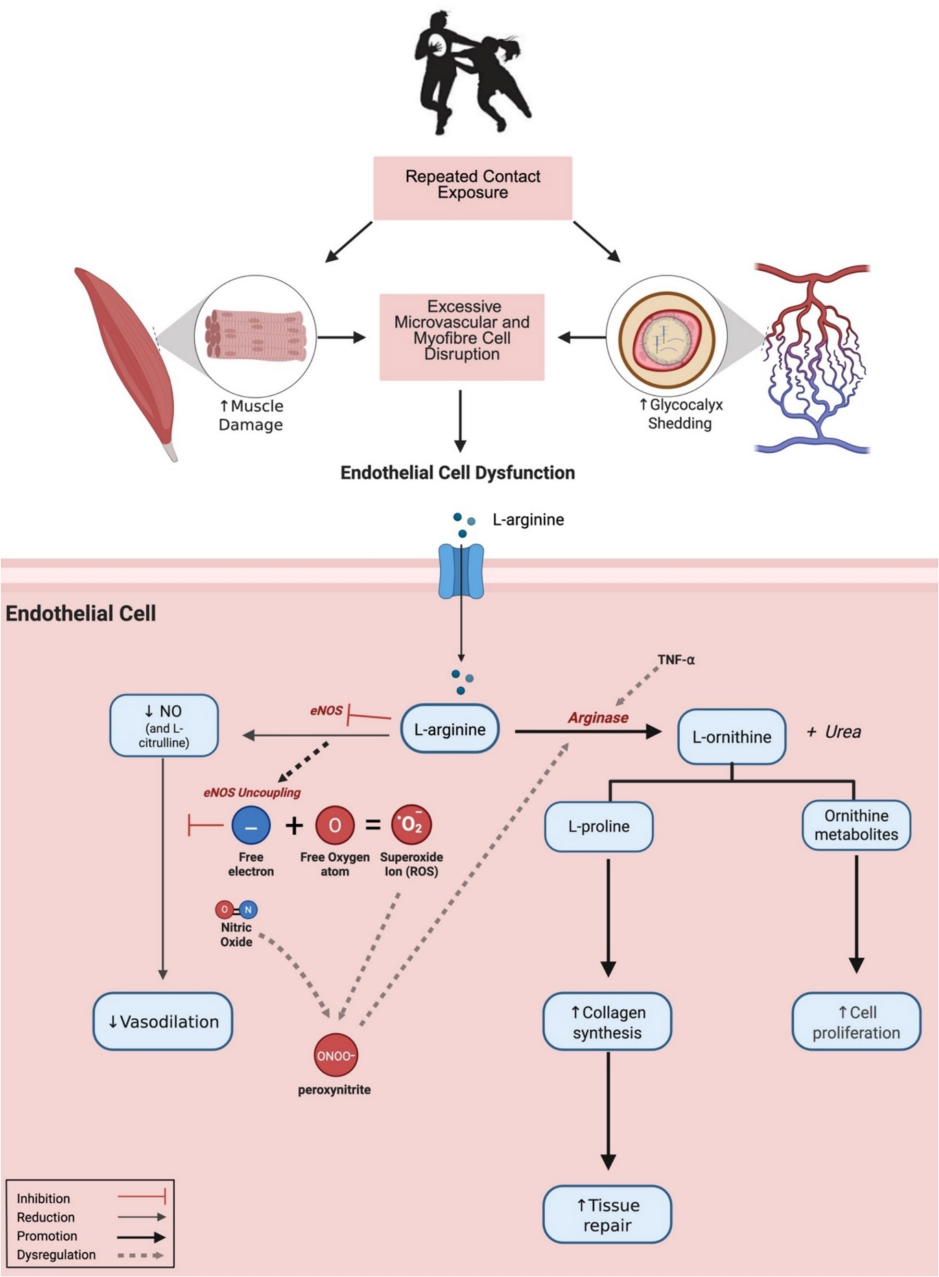
Proposed mechanism for vascular (endothelial) dysfunction following excessive impact-induced tissue damage (IITD): Upregulated arginase-1 reduces l-arginine availability for endothelial nitric oxide synthase (eNOS), resulting in decreased nitric oxide (NO) production and increased superoxide (O₂⁻) generation in the vascular endothelium. The excess O₂⁻ from uncoupled eNOS reacts with NO, forming peroxynitrite (ONOO⁻), which further amplifies arginase activity [159]. TNF-⍺ contributes to this process by upregulating arginase expression, shifting arginine metabolism from eNOS to arginase pathways [177, 178]. Increased arginase activity consumes more l-arginine, leading to further eNOS uncoupling and greater O₂⁻ production. This cascade of events leads to reduced NO bioavailability, impaired vasodilation and amplified oxidative stress, contributing to overall vascular dysfunction. Grey dashed lines represent pathways that may contribute to excessive tissue damage under conditions of high levels of IITD without adequate recovery. Created in BioRender. Bolger, C. (2025) https://BioRender.com/y99y354

One mechanism that could explain systemic endothelial dysfunction following TBI and other forms of tissue damage is the upregulation of arginase, which competes with endothelial nitric oxide synthase (eNOS) for their shared substrate, l-arginine [34]. Under these conditions, eNOS becomes uncoupled, donating electrons to molecular oxygen (O₂) rather than l-arginine, resulting in the production of superoxide $\left( {{\text{O}}_{{2}}^{ - } } \right)$, a reactive free radical that further depletes NO availability. The excess O₂⁻ from uncoupled eNOS reacts with NO, forming peroxynitrite (ONOO^−^), which further amplifies arginase activity [159]. Evidence from surgical trauma and TBI rodent models shows that tissue injury induces a marked increase in arginase expression and activity, coinciding with l-arginine depletion and reduced circulating NO [34, 35]. l-arginine serves as the primary substrate used in the synthesis of NO through eNOS-mediated oxidation. NO plays a central role in vasodilation, enhancing nutrient and oxygen delivery to working muscles, regulating vascular tone, and maintaining cardiovascular homeostasis [160, 161]. Prolonged reductions in NO bioavailability can reduce exercise tolerance, as well as invoke pathophysiological conditions such as endothelial dysfunction and arterial stiffness, both precursors of cardiovascular disease [161, 162] (Fig. 3).

The preferential oxidation of l-arginine by arginase could be explained by its role in wound healing and tissue repair. Arginase-related pathways intersect with the urea cycle, aiding in cell detoxification, proliferation, and collagen synthesis. These pathways play a key role in balancing inflammatory responses during wound healing [163]. Both local and systemic dysfunctions in arginase pathways have been linked to impaired healing and the development of chronic wounds following tissue trauma [163]. This sequence results in systemic endothelial dysfunction due to reduced NO production caused by arginase-induced degradation of l-arginine, which limits eNOS-dependent NO production and subsequent vasodilation [34]. This chain of events offers a potential mechanism by which tissue trauma from repeated contact exposure and head trauma in collision sports may lead to systemic vascular dysfunction, contributing to compromised recovery and long-term vascular health and player welfare issues in collision sport athletes [164–166].

Endothelial dysfunction, accelerated by the loss of NO, may also contribute to cognitive impairment [167]. Exposure to recurrent contacts and concussions in male rugby union players has been associated with accelerated cognitive decline, likely due to free radical-mediated reductions in vascular NO bioavailability [158, 168]. Although the specific mechanism(s) for reduced systemic NO bioactivity was not investigated, it is plausible that arginase-dependent uncoupling of eNOS contributes to the increase in free-radical production. Moreover, excessive shedding of eGC components might play a role in cognitive decline. Repeated contact involvements in collision sports could lead to the shedding of eGC components. Once in circulation, eGC components, such as heparan sulfate fragments, interact with soluble proteins implicated in cognitive impairment [169].

Collectively, repeated exposure to contacts (concussive and non-concussive) and the resulting IITD might contribute to excessive activation of arginase-dependent uncoupling of eNOS, increased eGC shedding, reduced NO, and increased free-radical production, and the health and performance implications that follow. Understanding the implications of IITD is crucial for both muscle tissue response and subsequent recovery, providing insights into the vascular response, recovery timelines and potential long term cardiovascular and cognitive implications for player welfare.

## Adaptation of Athletes to Exercise-Induced Muscle Damage Versus IITD

Muscle soreness after eccentric exercise can be attributed to muscle damage, disturbances in Ca^2+^ homeostasis, and/or activation of type IV nerve endings due to the by-products of infiltrating inflammatory cells [16]. The severity and time course of recovery depends on a number of variables including the number of contractions or eccentric actions performed, the intensity of those actions, muscle length, upper versus lower limb, single versus multiple joint actions, genetic variability and previous exposure to the same exercise [48]. Sex differences may also influence outcomes, with animal studies suggesting oestrogen protects muscle membranes and reduces inflammation [170]. Outcomes in human studies are less clear; however, it seems that the degree of neuromuscular fatigue and markers of muscle damage are more pronounced after exercise in men than in women [171, 172]. The most well-known factor that influences recovery from EIMD is previous exposure to muscle damage-inducing exercise, a phenomenon known as the ‘repeated bout effect’. Repeated exposure to exercise results in adaptations that diminish the associated symptoms when compared to initial exposures [48]. While the precise mechanism(s) behind this adaptation remain(s) unclear, it is likely that neural, mechanical and/or cellular changes regulate the inflammatory response and remodelling of the extracellular matrix involved in the repeated bout effect [173, 174]. It appears that the greatest protective adaptation in the repeated-bout effect occurs after the first unaccustomed bout of eccentric exercise, with additional bouts yielding minimal or no further benefit for either immediate strength preservation or recovery duration [45].

Rugby coaches often talk of an athlete being ‘contact fit’ or ‘contact ready’, implying that athletes may adapt to, or increase their tolerance to, contact exposure. Some researchers have postulated that like EIMD, IITD adaptations occur within skeletal muscle following repeated contact exposures analogous to the repeated bout effect [175]. This effect has since been coined ‘contact adaptation’ [4, 176]. Some preliminary research in support of this concept examined muscle damage markers throughout a competitive season of collegiate American Football [175]. CK levels were notably elevated after preseason camp but remained similar to baseline levels at all subsequent time points, possibly evidence of an adaptation to IITD. Pre-season training is typically more intense than the competitive season that follows. Moreover, after a period of relative inactivity like the off-season, athletes often experience EIMD due to the heightened training load during pre-season camps. Hence, the outcomes of this study may primarily reflect the well-established repeated-bout effect of EIMD rather than direct adaptations to contact [4].

If IITD is more like a contusion injury, prior exposure may not offer substantial protection against future occurrences. One possibility is that adaptation may not occur following contact exposure, in either muscle or vascular tissues. However, there may be a degree of desensitisation to repeated contact, leading to a reduced perception of muscle soreness. Contrary to this hypothesis, season-long monitoring in elite rugby league players revealed no attenuation in perceived muscle soreness across an entire competitive season, suggesting limited or absent physiological adaptation to the repeated contact exposure [83]. Future studies investigating ‘contact adaptation’ in relation to muscle and vascular damage, perceptual soreness, performance metrics such as sprint speed or jump height, and potential sex differences are warranted.

## Future Research

Future research is needed to first determine if there is a substantial relationship between participation in collision sports and IITD. Secondly, a drop-mass model should be utilised to explore isolated IITD, encompassing muscle and vascular response and its implications for health, performance and recovery. Thirdly, research should examine the concept of ‘contact fit’ or ‘contact adaptation’ and whether adaptation to IITD exists. Lastly, tackle-based investigation studies are likely needed to address this complex issue. A well-designed protocol that controls for non-contact-based efforts could offer a more ecologically valid approach for establishing a link between contacts and IITD. Additional methodological details are provided in OSM Resource 2.

## Conclusion

Current World Rugby guidelines give limited consideration to the physiological responses to contacts. While some studies identified positive associations between contact exposure and subsequent tissue damage, limited research has investigated isolated contact exposures and subsequent tissue damage. Contact events such as tackling, carrying into contact, rucks, scrums and mauls, along with secondary impacts with the ground and the subsequent impact of athletes falling onto the tackler or ball-carrier, may exert forces upwards of 4–5 kN or four to six times BM onto athletes. Repeated exposure to impact forces of this severity has the potential to cause damage to the tissue at or adjacent to the site of impact.

To encompass more than just muscle, the term impact-induced tissue damage or IITD has been suggested as a potential alternative to impact-induced muscle damage or IIMD. While EIMD and IITD may have commonalities in regeneration and remodelling processes, IITD appears to differ with an augmented inflammatory infiltrate and subsequent secondary damage response. If IITD is more akin to a contusion injury, then prior exposure to IITD may not provide a protective effect against future occurrences in the same manner that exercise does. Consequently, the cumulative effect of repeated contact exposure without adequate recovery could predispose collision sport athletes to excessive tissue damage. This sequence may lead to neurovascular impairments through mechanisms like endothelial dysfunction driven by excessive NO depletion, oxidative stress and eGC shedding. Collectively, these factors disrupt recovery and may adversely affect long-term cardiovascular health and cognitive function.

## Supplementary Information

Below is the link to the electronic supplementary material.

Online Resource 1: Computerised literature search strategies that were used for our PubMed search. (DOCX 18 KB)

Online Resource 2: Supplementary text describing an experimental set-up for assessing contact adaptation. (DOCX 105 KB)
