# Supplementary material for: Beyond the Hit: Muscle and Vascular Tissue Responses to Contact Exposure in Collision Sports—A Narrative Review
Source: Sports Med. 2025 Aug 17;55(11):2753–71. doi: 10.1007/s40279-025-02296-1 (PMC12559050; doi:10.1007/s40279-025-02296-1)
Supplement: Supplementary file 1 — Online Resource 1: Computerised literature search strategies that were used for our PubMed search. (DOCX 18 KB) [file 40279_2025_2296_MOESM1_ESM.docx]

Journal: Sports Medicine

Type: Narrative Review

# Title: Beyond The Hit: Muscle And Vascular Tissue Responses To Contact Exposure In Collision Sports – A Narrative Review

Short Title: Beyond The Hit

Authors:

Craig Bolger^1,2^, Jocelyn Mara^1^, David B. Pyne^1^, Andrew J. McKune^1,3^

Affiliations:

1. University of Canberra Research Institute for Sport and Exercise, Canberra, Australia
2. ACT Brumbies Rugby, Canberra, Australia
3. School of Health Sciences, Biokinetics, Exercise and Leisure Sciences, University of KwaZulu-Natal, Durban, KZN, South Africa.

Address for Correspondence:

Craig Bolger

University of Canberra Research Institute for Sport and Exercise

Kirinari Street, Bruce, ACT, 2617

Australia

[Craig.bolger@canberra.edu.au](mailto:Craig.bolger@canberra.edu.au)

**Supplementary material 1 - PubMed Search Queries**

("Rugby" OR "Rugby Union" OR "Rugby League" OR “Rugby Sevens”) AND ("tackle*" OR "collision*" OR "contact sport*" OR “Contact” OR "impact") AND ("tissue damage" OR "muscle damage" OR "exercise-induced muscle damage" OR "EIMD" OR "Impact-Induced Muscle Damage" OR "IIMD" OR "inflammation" OR "biomarkers" OR "Creatine Kinase" OR "CK" OR "myoglobin" OR "C-Reactive Protein" OR "psychophysiological response" OR "neuromuscular fatigue" OR "neuromuscular performance" OR "countermovement jump" OR "CMJ" OR "Isokinetic Dynamometry" OR "Strength testing" OR "subjective questionnaire" OR "subjective readiness" OR " subjective soreness" OR "Wellness Questionnaire" OR "recovery" OR "fatigue" OR “Stress”)

("Rugby" OR "Rugby Union" OR "Rugby League" OR "Rugby Sevens") AND ("tackle*" OR "collision*" OR "contact sport*" OR "Contacts" OR "impacts") AND ("tissue damage" OR "muscle damage" OR "exercise-induced muscle damage" OR "EIMD" OR "Impact-Induced Muscle Damage" OR "IIMD" OR "inflammation" OR "biomarkers" OR "Creatine Kinase" OR "CK" OR "myoglobin" OR "C-Reactive Protein" OR "psychophysiological response" OR "neuromuscular fatigue" OR "neuromuscular performance" OR "countermovement jump" OR "CMJ" OR "Isokinetic Dynamometry" OR "Strength testing" OR "subjective questionnaire" OR "subjective readiness" OR " subjective soreness" OR "Wellness Questionnaire" OR "recovery" OR "fatigue" OR "Stress") AND ("female" OR "Woman" or "Women's" OR "Girls") NOT ("Concussion")

("Rugby" OR "Rugby Union" OR "Rugby League" OR "Rugby Sevens" OR "contact sport*" ) AND ("vascular response" OR "microvascular" OR "vascular damage" OR "endothelial dysfunction" OR "glycocalyx" OR "cardiovascular")

("Rugby" OR "Rugby Union" OR "Rugby League" OR "Rugby Sevens" OR "contact sport*" ) AND ("Contusion Injury" OR "muscle injury" OR "drop-mass" OR "IIMD" OR "Impact-Induced Muscle Damage")

**("Contusion Injury" OR "muscle injury" OR "IIMD" OR "Impact-Induced Muscle Damage") AND ("drop-mass Model")**

("Contusion Injury" OR "IIMD" OR "Impact-Induced Muscle Damage") NOT ("Spinal")
