# Supplementary material for: Beyond the Hit: Muscle and Vascular Tissue Responses to Contact Exposure in Collision Sports—A Narrative Review
Source: Sports Med. 2025 Aug 17;55(11):2753–71. doi: 10.1007/s40279-025-02296-1 (PMC12559050; doi:10.1007/s40279-025-02296-1)

Journal: Sports Medicine

Type: Narrative Review

# Title: Beyond The Hit: Muscle And Vascular Tissue Responses To Contact Exposure In Collision Sports – A Narrative Review

Short Title: Beyond The Hit

Authors:

Craig Bolger^1,2^, Jocelyn Mara^1^, David B. Pyne^1^, Andrew J. McKune^1,3^

Affiliations:

1. University of Canberra Research Institute for Sport and Exercise, Canberra, Australia
2. ACT Brumbies Rugby, Canberra, Australia
3. School of Health Sciences, Biokinetics, Exercise and Leisure Sciences, University of KwaZulu-Natal, Durban, KZN, South Africa.

Address for Correspondence:

Craig Bolger

University of Canberra Research Institute for Sport and Exercise

Kirinari Street, Bruce, ACT, 2617

Australia

[Craig.bolger@canberra.edu.au](mailto:Craig.bolger@canberra.edu.au)

**Supplementary material 2.0 – Proposed experimental set-up for assessing contact adaptation.**

Using a drop-mass model as described in section 2.3.4, initial investigations should employ repeated contact exposures over multiple sessions separated by 1-2 weeks. These studies should quantify changes in muscle and vascular damage via blood biomarkers and imaging techniques. Vascular responses can be assessed non-invasively using devices like laser Doppler flowmetry, ultrasound imaging, or near-infrared spectroscopy to characterise tissue perfusion and blood flow dynamics. Additionally, psychological responses and neuromuscular performance metrics, such as contractile kinetics, jump tests, or strength testing, should be evaluated to determine if protective physiological responses emerge over time.

To complement laboratory, a well-designed tackle contest protocol that controls for non-contact-based efforts usings an 8 x 8 meter grid, were participants would execute full-intensity tackles without contesting the ball after the tackle is completed. Such a setup would allow for simulation of real-life contact scenarios, where players are required to execute tackles from a moving start, while minimizing the risk of EIMD from high-speed running actions, offering a more ecologically valid approach for establishing a link between contact exposure and IITD compared to a drop-mass model.

To explore the concept of ‘*contact adaptation’* in realistic contexts, participants should be randomly allocated into one of two groups over a structured one- to two-week training window. One group would participate in two additional contact-specific training sessions each week, while the second group would engage in equivalent non-contact conditioning and training activities. Following the training period, all participants would repeat baseline assessments, including physiological testing and the standardized tackle protocol. This controlled exposure design permits a direct comparison of adaptations between groups, enabling practitioners to identify whether repeated contact exposure leads to protective physiological adaptations against IITD in a real-world scenario.


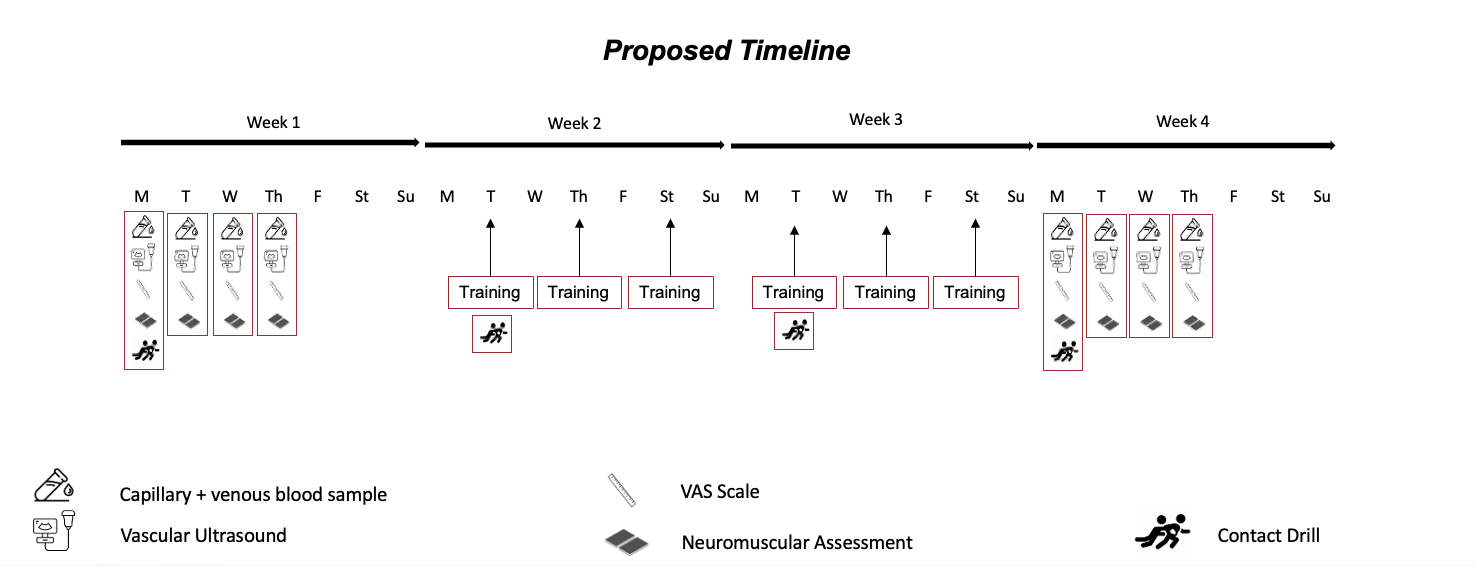

Supplement: Supplementary file 2 — Online Resource 2: Supplementary text describing an experimental set-up for assessing contact adaptation. (DOCX 105 KB) [file 40279_2025_2296_MOESM2_ESM.docx]
